# Sera From Patients With Minimal Change Disease Increase Endothelial Permeability to Sodium

**DOI:** 10.1016/j.ekir.2020.04.010

**Published:** 2020-04-20

**Authors:** Florence Daviet, Muriel G. Blin, Karim Fallague, Richard Bachelier, Manon Laforêt, Manon Carré, Stéphane Poitevin, Françoise Dignat-George, Marcel Blot-Chabaud, Nathalie Bardin, Stéphane Burtey, Noémie Jourde-Chiche, Aurélie S. Leroyer

**Affiliations:** 1 Aix-Marseille University, Centre de recherche en CardioVasculaire et Nutrition, Institut national de la santé et de la recherche médicale 1263, Institut national de recherche pour l’agriculture, l’alimentation et l’environnement 1260, Marseille, France; 2 Assistance Publique-Hôpitaux de Marseille, Centre de Néphrologie et Transplantation Rénale, Hôpital de la Conception, Marseille, France; 3 Aix-Marseille University, Centre de Recherche en Oncologie Biologique et Oncopharmacologie, Institut national de la santé et de la recherche médicale unité mixte de recherche 911, Marseille, France; 4 Assistance Publique-Hôpitaux de Marseille, Laboratoire d’Immunologie, Hôpital de la Conception, Marseille, France

Edema is the main symptom of nephrotic syndrome associated to minimal change disease (MCD). Besides the increase in glomerular permeability, possibly induced by a circulating permeability factor,^1^ an increase in systemic vascular permeability could participate in the constitution of edema.

This study was conducted to evaluate the permeability of endothelial cells (EC) exposed to serum from nephrotic patients with MCD, to low- or high-molecular-weight molecules, by trans- or paracellular transport.

## Results

### MCD Serum Induces an Increase in Human Umbilical Vein Endothelial Cell Permeability *In Vitro*

**Figure 1:**
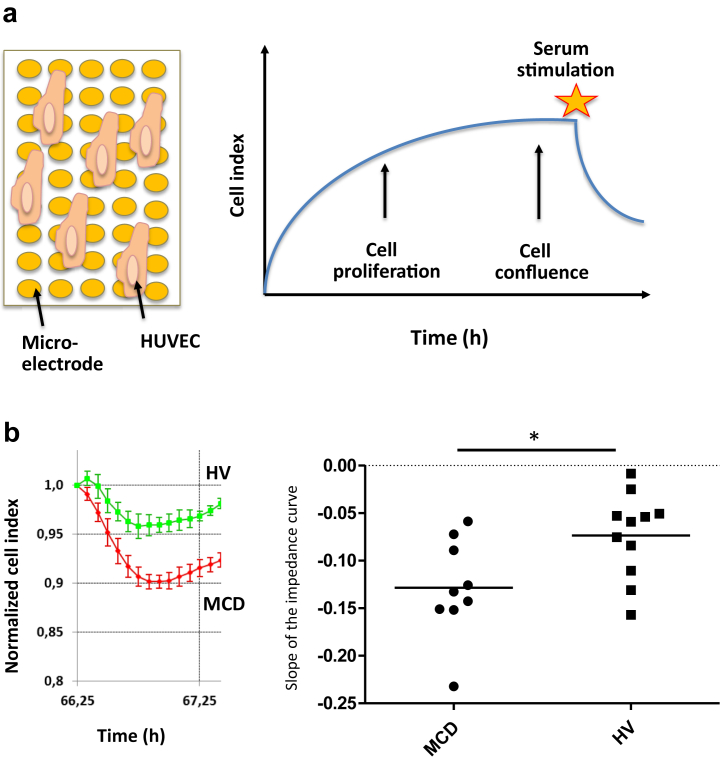
Sera from minimal change disease (MCD) patients increase human umbilical vein endothelial cell (HUVEC) permeability evaluated by impedance *in vitro*. (a) The xCELLigence Real-Time Cell Analyzer system (ACEA Biosciences, Inc., San Diego, CA) allows the evaluation of the permeability of a monolayer of endothelial cells (i.e., HUVECs), seeded in the wells of an electronic microtiter plate. The cellular impedance is measured from the beginning of HUVEC culture, and HUVECs are stimulated with sera once they have reached confluence. (b) Results obtained after stimulation of confluent HUVECs by MCD patient sera (n = 10) and healthy volunteer (HV) sera (n = 11). The steeper slope observed with MCD compared to HV sera reflects an increased permeability of HUVEC monolayer. The slopes of impedance curves were measured between T0 and T45 minutes from the beginning of stimulation with sera. The MCD patient sera induce a greater permeability than the HV sera. ∗*P* = 0.0276.

First, we evaluated the effect of MCD serum on the global permeability of human umbilical vein endothelial cells (HUVECs) via the xCELLigence Real Time Cell Analyzer system (ACEA Biosciences, Inc., San Diego, CA) (Figure 1a). We observed a significant decrease in the impedance, reflecting an increased permeability, with sera from MCD patients compared to sera from healthy volunteers (HVs) (Figure 1b).

**Figure 2:**
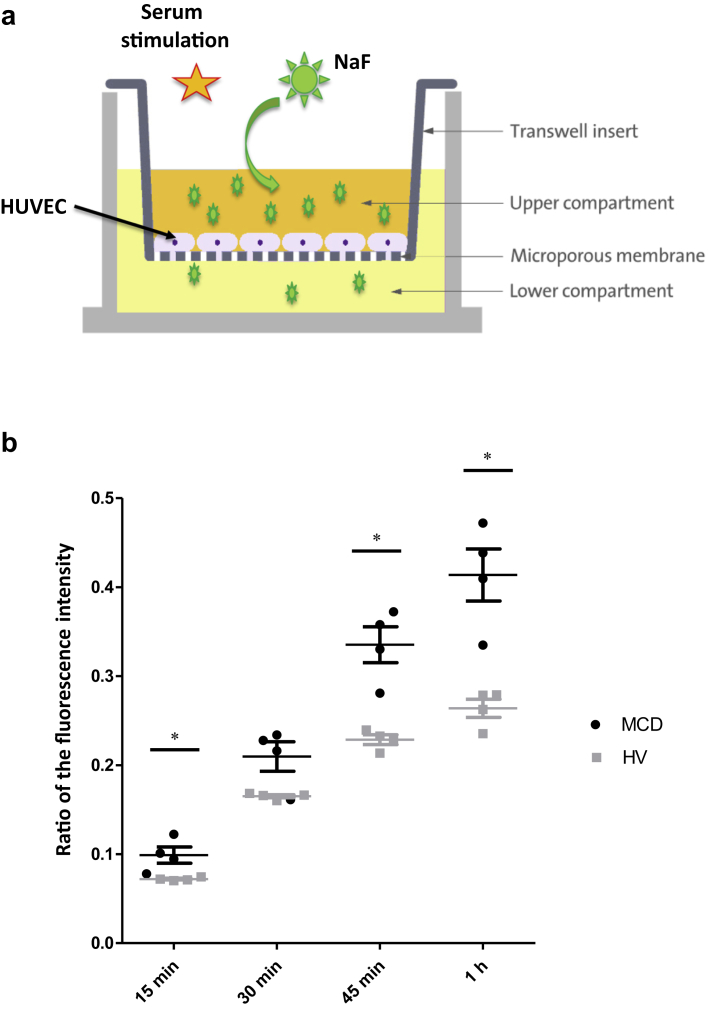
Sera from minimal change disease (MCD) patients increase human umbilical vein endothelial cell (HUVEC) permeability to sodium *in vitro*. (a) The passage of sodium fluorescein (NaF, 276 Da) is measured across a semipermeable membrane from the Transwell system at different times (15, 30, and 45 minutes and 1 hour) after stimulation of confluent HUVECs by patient sera. (b) Results are expressed as the ratio of the fluorescence intensity between the lower and upper chambers of the Transwell system (Thermo Fisher Scientific, Waltham, MA). More passage of NaF was observed at T15, T45, and T60 minutes after stimulation with MCD patient sera (n = 5) compared to healthy volunteer (HV) sera (n = 5). ∗*P* < 0.05.

Second, we compared the endothelial permeability, via the Transwell system (Thermo Fisher Scientific, Waltham, MA), for FITC-Dextran, a high-molecular-weight (HMW) molecule (40kDa), and for sodium fluoresceine (NaF), a low-molecular-weight (LMW) molecule (276 Da) (Figure 2a). No difference was observed between MCD patient and HV sera in terms of passage of FITC-Dextran (Supplementary Figure S1). Conversely, the passage of a NaF through the HUVEC monolayer was significantly greater at 15, 45, and 60 minutes after stimulation with MCD patient sera compared to HV sera (Figure 2b).

We therefore demonstrated an increased permeability of HUVEC *in vitro* after stimulation by sera of patients with MCD, probably restricted to LMW molecules such as sodium.

### Paracellular Permeability Is Not Affected by MCD Patient Serum

We evaluated *in vitro*, by western blot analysis, the quantity of vascular endothelial (VE)−cadherin (total and phosphorylated), reflecting paracellular permeability (tight junctions). No difference was observed between MCD patient and HV sera (Supplementary Figure S2A and B).

We then evaluated *in vivo* the paracellular permeability induced by the sera of patients, injected in the skin of mice, using the Miles Assay technique. The extravasation of Evans blue dye, a molecule strongly bound to albumin, did not differ between skin zones injected with MCD patient and HV sera (Supplementary Figure S2C).

### Transcellular Permeability of HUVECs Is Increased by MCD Patient Serum

#### Without Involvement of Caveolin 1

The quantity of Caveolin 1 was not different in lysates of HUVECs stimulated with MCD patient or HV sera in western blot analysis (Supplementary Figure S3A and B).

The transfection of HUVEC with small interfering ribonucleic acids (siRNAs) targeting Caveolin 1 decreased the expression of this protein compared to control siRNA (Supplementary Figure S3C) but did not modify the increased permeability to NaF after stimulation with MCD patient sera.

#### With a Role of Endothelial Sodium Channel Activation by Serine Proteases

**Figure 3:**
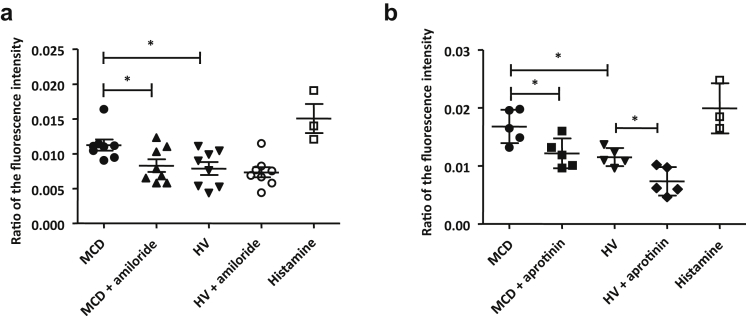
The increased permeability of human umbilical vein endothelial cells (HUVECs) to sodium is reversed by amiloride and by aprotinin. (a) Measurement of the sodium fluorescein (NaF) passage through a semipermeable membrane of the Transwell system (Thermo Fisher Scientific, Waltham, MA) after stimulation of HUVECs by sera from minimal change disease (MCD) patients or healthy volunteers (HVs), with or without addition of amiloride (1 μM), 15 minutes after the start of stimulation. The results are expressed as the ratio of the fluorescence intensity between the lower and upper chambers. The increased permeability of HUVECs to NaF induced by MCD patient sera is reversed by amiloride (*P* = 0.0499). Amiloride has no effect on HUVEC permeability after stimulation with HV sera. ∗*P* < 0.05. (b) Measurement of the sodium fluorescein (NaF) passage through a semipermeable membrane of the Transwell system after stimulation of HUVEC by sera from MCD patients or HV, with or without adjunction of aprotinin (100 μg/ml), 15 minutes after the start of stimulation. The increased permeability of HUVEC to NaF induced by MCD patient sera is reversed by aprotinin (*P* = 0.0317). Aprotinin also reduced the permeability of HUVECs after stimulation with HV sera. ∗*P* < 0.05.

We evaluated the role of endothelial sodium channels in the increased permeability due to MCD patient sera. The HUVECs were incubated in the Transwell system with MCD or HV sera with or without amiloride, an inhibitor of sodium channels such as ENaC (Figure 3a). The increase in NaF permeability induced by MCD patient sera was significantly reversed by amiloride (*P* = 0.0499).

We then evaluated the role of serine proteases in the increased endothelial permeability due to MCD patient sera. The HUVECs were incubated with MCD patient or HV sera with or without aprotinin, an inhibitor of serine proteases (Figure 3b). The increase in NaF permeability induced by MCD patient sera was reversed by aprotinin (*P* = 0.0465). In addition, the baseline endothelial permeability of endothelium with HV sera was reduced with aprotinin.

To determine whether the channel involved in this increased endothelial permeability to sodium was the endothelial ENaC, we studied ENaC subunits using western blot analyses. We did not detect any differences with MCD patient or HV sera in the cleavage of the ENaC-α, -β, or -γ subunits (Supplementary Figure S4A−C).

## Discussion

In the present study, we show that serum from MCD patients increases the permeability of cultured EC to sodium through a transcellular pathway involving a sodium channel inhibited by amiloride and aprotinin.

Edema is a major clinical symptom in patients with MCD, favored both by decreased oncotic pressure (hypoalbuminemia) and by increased renal sodium reabsorption (activation of tubular ENaC in the collecting duct), and possibly by still-unidentified factors.^2^ Indeed, a permeability factor in MCD has been sought for decades,^1^ but mainly to determine the cause of albumin leakage through the glomerular filtration barrier,^1^^,^^3^ whereas we propose here a role of systemic endothelial permeability to explain the brutal constitution of edema. In fact, we demonstrate that MCD patient sera increase endothelial permeability to sodium, thus allowing a large passage of sodium and fluid in the interstitial space.

One of the major roles of the vascular endothelium is to provide a selective barrier between blood circulation and tissue. This selective permeability is controlled by intercellular junctions (tight and adherent junctions, regulating paracellular permeability), transport through the endothelium (transcellular permeability), and interactions between EC and pericytes.^4^ The transcellular permeability involves caveolae, which are invaginations of the plasma membrane structured mainly around caveolin-1; transcellular pores or channels allowing passage of LMW solutes; and vesiculovacuolar organelles.^5^ In this work, we show that only transcellular permeability is affected by MCD patient serum, and exclude a role of caveolin 1.

We demonstrate an increase in vascular permeability to sodium, which is reversed by amiloride, and by aprotinin. Some animal and human data support the use of amiloride, as an inhibitor of ENaC, in nephrotic patients.^6^, ^7^, ^8^ ENaC is a sodium channel mainly expressed by the distal and collecting tubule, which is activated after the cleavage of its α- and γ-subunits by serine proteases such as plasmin.^8^ In nephrotic syndrome, an aberrant filtration of blood serine proteases leads to an increased urinary excretion of these proteases and exposure of ENaC to their proteolytic properties, with increased ENaC activation and sodium reabsorption.^8^ A high serine protease activity is observed in the urine of nephrotic patients,^8^ which resolves after remission, and the fluid overload of nephrotic patients is correlated with their urinary excretion of serine proteases.S1 Moreover, the systemic administration of aprotinin, a protease inhibitor, prevents the formation of edema in nephrotic mice, despite a similar level of proteinuria.^9^

ENaC, in addition to its renal localization, is also expressed in the endothelium, where it is involved in morphological and mechanical properties.^3^^,^S2^,^S3 Here, although we show that both amiloride and aprotinin reverse the increase in salt permeability of ECs exposed to nephrotic serum, we observe no differential cleavage of ENaC by western blot. These results do not rule out a role for ENaC in the endothelial permeability to sodium induced by MCD patient serum. In particular, the use of undiluted serum (instead of 1/10 diluted serum) would have been closer to the *in vivo* conditions and may have revealed a differential cleavage of ENaC by western blot. However, ENaC is not the only sodium channel inhibited by amiloride.S4 Another hypothesis is that another endothelial sodium channel, also inhibited by amiloride and activated by a serine protease present in the serum of healthy controls and increased in the serum of MCD patients, could be implicated in this increased endothelial permeability.

In nephrotic patients, the benefit of amiloride could be related not only to its diuretic properties but also to the reduction of endothelial salt permeability and fluid leakage. Our work also suggests that the baseline physiological activity of endothelial sodium channels could be maintained by serine proteases, as HUVEC permeability to sodium was reduced in the presence of aprotinin.

There are several limitations to our study. First, because of the limited amount of serum available for each patient, we used diluted sera in all experiments, and this may have blunted the effect of MCD patient serum. Future research in the field may benefit from the use of undiluted, or less diluted, serum. Second, our experiment model based on HUVECs does not include the pericytes or vascular wall, which also participate in vascular permeability. Yet, because edema probably arise from capillary leaks, a monolayer of HUVECs may be a relevant model in this setting. Third, only MCD patient sera were tested, without nephrotic or non-nephrotic controls from patients with other kidney diseases, and the specificity of this endothelial permeability cannot be asserted. Finally, we have not identified *the permeability factor* of MCD, or the sodium channel that it activates. However, this work highlights the fact that the permeability factor of MCD may not be solely a glomerular one (allowing the leakage of albumin through the glomerular filtration barrier), but also a systemic one, allowing the leakage of sodium in the interstitial space, possibly through the activation of an endothelial sodium channel by proteases.

In conclusion, this original work shows, for the first time, an increased endothelial permeability induced by sera from patients with MCD, allowing the passage of sodium through the endothelium, probably participating in the constitution of edema, and reversed by amiloride and by aprotinin.

## Disclosure

All the authors declared no competing interests.

## Supplementary Material

Supplementary File (Word)
