# Supplementary material for: Sera From Patients With Minimal Change Disease Increase Endothelial Permeability to Sodium
Source: Kidney Int Rep. 2020 Apr 20;5(7):1071–5. doi: 10.1016/j.ekir.2020.04.010 (PMC7335967; doi:10.1016/j.ekir.2020.04.010)
Supplement: Supplementary File (Word) [file mmc1.docx]

**Online supplemental material**

**Material and methods**

1. **Patients, preparation and conservation of sera**

The sera of nephrotic patients with MCD were sampled at the time of renal biopsy, after signature of a written informed consent, and included in the biocollection DC-2012-1704 (Laboratory of Immunology, AP-HM, Marseille), with corresponding clinical and biological data (**Supplemental Table 1**). Sera from age- and sex-matched healthy volunteers (HV) were also tested. Blood collection of all patients and HVs was performed on a dry tube which, after incubation for at least 1 hour at room temperature, was centrifuged at 3000 rpm for 10 minutes. After centrifugation, the serum was collected, aliquoted and stored at -80 ° C.

1. **Endothelial cells culture**

Human Umbilical Vein Endothelial Cells (HUVEC) were derived from human umbilical cord veins from term births, collected at the maternity ward of the Hôpital de la Conception of the University Hospital of Marseille. They were isolated according to the method of Jaffe [S5].

The cells were cultured after seeding on different culture media previously coated with 0.2% gelatin (porcine gelatin, Sigma-Aldrich, Saint Quentin Fallavier, France). They were cultured under standard conditions (humidified atmosphere, 37 ° C., 5% CO2), in a specific medium, EGM2 (Endothelial Growth Medium, Lonza, Basel, Switzerland), comprising growth factors (hFGF, hEGF, VEGF, IGF1), hydrocortisone, vitamin C, heparin, gentamycin, penicillin, streptomycin, amphotericin B, amino acids and fetal calf serum (FCS) at 2%. The cells were used from the second to the fifth passage.

1. **Stimulation of HUVEC with sera**

After inoculation of a variable number of cells according to the support of culture used, the HUVEC were maintained for 48 hours in the incubator until a monolayer of confluent cells was obtained. The standard culture medium (EGM2) was then replaced by a deprived medium consisting of EBM2 (Endothelial Basal Medium, Lonza, Basel, Switzerland) containing 1% fetal calf serum (FCS). After a period of deprivation of 18 hours, the cells were stimulated for 1 hour by the serum of the patients or healthy volunteers (1/10 diluted). T0 was the beginning of this stimulation. Deprivation medium alone or containing FCS (1/10) was used as a negative control; recombinant human TNF alpha (PeproTech, USA) at 20 ng / ml, known to induce vascular permeability, was used as a positive control. Amiloride (Sigma A7410) was also incubated at a concentration of 1µM during 1 hour before the exposure of HUVECs to sera.

1. **Measurement of HUVEC monolayer permeability by impedance**

The permeability of HUVEC, namely their ability to maintain an intact barrier, was analyzed using the xCELLigence Real-Time Cell Analyzer (ACEA Bioscience / Roche Applied Science). This technology [S6] measures the electrical impedance of cells cultured on microelectrodes in a 96-well plate. This cell impedance can be used to estimate the integrity of a monolayer of cells once they have reached confluence, and this, steadily over several days. A decrease in cell impedance (modeled by the slope of the curve described by the impedance signal) reflects an increase in para- or trans-cellular permeability [S7].

HUVEC were cultured for 48 hours at a density of 50,000 cells per well in a E-Plate 96 (Roche Applied Science) previously coated with 0.2% gelatin. The impedance was measured every 15 minutes: it gradually increased as cell proliferation progressed and then reached a plateau when the cells reached confluence.

Once the confluence had been obtained, HUVEC were deprived for 18 hours and then stimulated as previously described, after verifying the stability of the impedance curves (cell viability). Each stimulation condition was performed in triplicate.

After stimulation, the impedance was measured every 5 minutes for 6 hours. The induced permeability was modeled according to a standardized method: normalization of the curves at the time of the stimulation, measurement of the slope between the beginning of the stimulation and the 45th minute, and analysis of the curves.

1. **Endothelial transmigration assay**

To measure *in vitro* endothelial permeability to molecules of high molecular weight induced by sera of patients or HV, HUVECs (80,000 cells per well) were cultured on 24-well plates associated with a Transwell system [S8] consisting of polycarbonate inserts (Diameter: 6.5mm, pore diameter: 3μm, Falcon). After 48 hours of incubation, when a monolayer of confluent endothelial cells had been obtained, the cells were deprived for 18 hours. The culture medium of the upper chamber was then replaced by EBM2 containing 1% FCS and FITC-Dextran (40 kDa, Sigma Aldrich) at 0.5 μg/mL. The cells were afterwards stimulated by MCD or HV sera as previously described, each stimulation condition was performed in duplicate. The passage of FITC-Dextran through the semipermeable membrane was evaluated by taking 50 μL of medium, after homogenization thereof, in the lower chamber after 0, 15, 30, 45 minutes and 1 hour of stimulation. After each sample had been taken from the lower chamber, 50 μL of deprivation medium was added in order to maintain equal volumes in both chambers throughout the experiment. Fluorescence was measured using the Infinite iTecan reader (excitation wavelength: 485 nm, emission wavelength: 535 nm).

We also measured the permeability of a HUVEC monolayer to low molecular weight molecules. The protocol was similar to that described above, but the Transwell system used was a support of 6.5 mm in diameter with smaller pores of 0.4 μm in diameter, the low molecular weight molecule used was Sodium Fluorescein (NaF, 376 Da, Sigma Aldrich) at a concentration of 10 μg/mL. The samples in the lower chamber were made at the same time points but were 10 µL samples and were diluted to the tenth before reading.

1. **Si-RNA of Caveolin 1**

Transfection with human Caveolin 1 si-RNA was carried out in order to abolish the expression of Caveolin 1. HUVEC were seeded in 6-well plates and transfected 24 hours after inoculation with either a control siRNA (SignalSilence Control siRNA ♯6568, Cell Signaling), or with a mixture containing three types of Caveolin 1 siRNA (s2448, s2447, s2446, Ambion, Life Technologies) blocking in different places the mRNA of Caveolin 1. The transfection was carried out by magnetofaction using SilenceMag beads (OZ Biosciences, France) according to the manufacturer's recommendations. During magnetofaction and for the next 6 to 8 hours, HUVEC were cultured in OPTI-MEM (Reduced Serum Medium, Gibco, Life Technologies). 48 hours after the magnetofaction, HUVEC were seeded in the Transwell wells at a concentration of 67,000 cells per well. 96 hours after the magnetofaction, HUVEC were deprived according to the previously described protocol. The decrease in the expression of Caveolin 1 at 72 and 96 hours was verified in RT-PCR for the mRNA and in Western Blot for the protein.

1. **Western Blot analyses**

Several proteins of interest were sought and quantified by Western Blot from protein extracts derived from HUVEC lysates: the cells were washed with PBS (Phosphate Buffer Saline) containing calcium and phosphorus, then scraped in the presence of lysis buffer (Tris 1M pH8, 0.5M EDTA pH8, NaCl 5N, Nonidet P40 10%), combined with a protease and phosphatase inhibitor (1 pellet, Pierce, Thermo Scientific) at cold temperature. The lysis buffer containing the cells was then stirred at 4°C for 30 minutes and centrifuged for 5 min at 14000 g, the supernatant was then collected, aliquoted and stored at -80°C.

A protein assay was carried out using the Pierce BCA protein assay kit (Thermo Scientific) according to the supplier's instructions, in order to prepare samples containing 30 μg of proteins diluted in 30 μl of lysis buffer, for each condition.

The samples were loaded with a loading buffer under reducing conditions (LDS Sample Buffer (4X) and Sample Reducing Agent (1X), NuPAGE, novex, Life Technologies) and then boiled for 5 minutes at 90 ° C. The samples, along with a molecular weight marker (Spectra Multicolor Broad Range Protein Ladder, Thermo Scientific), were then placed in the wells of a 4-12% polyacrylamide gel (NuPAGE Tris-Acetate Mini Gels, Life Technologies) placed in an Invitrogen tank filled with migration buffer (The NuPAGE MOPS SDS Running buffer: MOPS, Tris base, SDS, EDTA and water); the migration was carried out for 1 hour at 150 V.

The liquid transfer was then carried out on a 0.45 μm nitrocellulose membrane (Amersham Protran, GE Healthcare Life Science) in the XCell II Blot module filled with transfer buffer (Tris base, glycine, TBS and water, to which is added methanol to 20%) for 2 hours at 33 V.

After transfer, the membrane was incubated for 1 hour in saturation buffer (0.1% TBS Tween 20 with 5% human serum albumin (BSA)). The incubation of the diluted primary antibody in saturation buffer was then carried out for 18 h at 4°C with stirring: anti-total VE cadherin antibody diluted 1/50 (120 kDa, ab7047, abcam), anti-phosphorylated VE cadherin on Tyr658 diluted 1/1000 (120 kDa, AB1955 Merck Millipore), anti-Caveolin 1 diluted 1/1000 (21kDa, D46G3 Rabbit mAb, Cell Signaling Technology), anti ENac-α/β/γ diluted 1/500 (Rabbit mAb, Stessmarq Biosciences), anti-β actin diluted 1/3000 (45kDa, D6A8 ♯8457, Cell Signaling Technology). The incubation of the secondary antibody diluted 1/5000 in saturation buffer was then carried out for 2 hours in ambient air with stirring: Goat anti-mouse IgG HRP (Horseradish peroxidase) antibody (Pierce, Thermo Scientific) and Goat anti -rabbit IgG HRP antibody (Pierce, Thermo Scientific). Each of these steps was preceded by several washing steps with wash buffer (0.1% TBS Tween 20).

Revelation was made by chemo luminescence using the Pierce ECL Western Blotting Substrate Kit (♯3236, Thermo Scientific) or the West Femto SuperSignal Kit (♯34096, Thermo Scientific), and images were captured by the GBOX Imager (Syngene). The intensity of the bands was quantified using GeneTools software (Syngene).

1. **Animals**

Female wild type mice C57BL/6 aged from 6 to 12 weeks were used to perform the in vivo experiments. Each experiment on animals was carried out with the prior agreement of the Ethics Committee for Animal Experimentation of Marseille and the Ministry of Higher Education and Research (n°00781).

1. **Measurement of vascular permeability in vivo through the Miles Assay**

For the Miles assay [S9], the mice were anesthetized by intraperitoneal injection of a mixture of: Xylasin 5% (Rompun 2% solution for injection, Bayer), Ketamine 15% (Ketamine 50 mg / ml Virbac France), and Atropine 1.25% (Atropine 1 mg / ml, Aguettant) diluted in saline, the dose injected being adapted to the weight of the animal. The mice were placed on a plate thermostated at 37 ° C in order to maintain the physiological temperature of the animal throughout the experiment.

Once the general anesthesia of the animal was obtained, an intravenous injection by retro-orbital route of Evans Blue was carried out: injection of 150 μL of a solution of Evans Blue 1% (Evans Blue, Sigma) diluted in PBS without calcium or phosphorus. The back of the mouse was shaved. 3 minutes after injection of Evans Blue, 40 μL intra-dermal injections were performed on the back of the mouse, with 6 injections per mouse: a positive control consisting of histamine at 1 μg / ml, a negative control constituted by PBS - / - and an injection of patient serum and a paired HV duplicate. 20 minutes after the end of the intradermal injections, the mouse was euthanized, the skin of the back was dissected and spots of skin of identical size were taken and placed in 500 μL of Formamide (Prolabo, RP Normapur, AR). The spots were incubated in Formamide for 18 hours at 55 ° C, then the supernatant was recovered and the optical density was measured at 630 nm with the microplate photometer (Multiskan acent, Thermo Scientific).

1. **Statistical Analyses**

The calculations of statistical significances between the various conditions were performed using the Mann-Whitney nonparametric U-tailed test. The results were considered statistically significant for p-values (p) less than 0.05. Graphs and statistical analyzes were performed with GraphPad Prism software (GraphPad software, San Diego, CA).

**Supplemental Table 1: Patient’s characteristics**

|  | **Minimal Change**  **Disease** | **Healthy**  **Volunteers** |
| --- | --- | --- |
| Number | 18 | 15 |
| Age, mean (range) | 42.1 (16-59) | 35.9 (26-53) |
| Gender (M/F) | 11/7 | 66.7 / 33.3% |
| Serum Albumin (g/dL), mean  Proteinuria (g/day), mean | 1.96  8.5 |  |
| Serum creatinine (mg/dL), mean | 1.48 |  |
| Important edematous syndrome | 72.2% |  |

**Supplemental Figures**

**Supplemental Figure 1: Sera from MCD patients do not modify the permeability of HUVEC to high molecular weight molecules *in vitro***

We measured the passage of FITC-Dextran (40 kDa) across a semi-permeable membrane (pore size: 3 μm for the FITC-Dextran experiment) from the Transwell system at different times (15, 30, 45 minutes and 1 hour) after the start of the stimulation of HUVEC by patient sera. The results are expressed as the ratio of the fluorescence intensity between the upper and the lower chambers.

Results are shown for 5 MCD patients and 5 HV. The passage of FITC-Dextran was not statistically different when HUVEC were stimulated by MCD or HV sera (p=0.84)


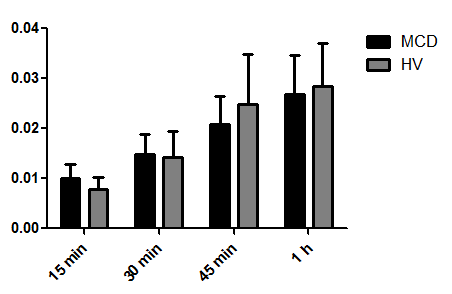


Ratio of the fluorescence intensity

**Supplemental Figure 2: The increase in HUVEC permeability is not due to paracellular pathway modifications**

**A:** Western blot performed on HUVEC cell lysates after 1 hour of stimulation by the sera of MCD or HV patients, or of recombinant human TNF used as a positive control (20 ng / ml)

**B :** Semi-quantitative measurement of the expression of different proteins by Gene Tools software (Syngene). p=0.49

**C :** Measurement of vascular permeability in vivo by intravenous injection of Evans Blue to 4 female C57BL / 6 mice, followed by intradermal injections of: sera of MCD patients and HVs in duplicate, PBS - / - as negative control and Histamine 1 μg / ml as positive control. Measurement of extravasation of Evans Blue after mouse euthanasia at the cutaneous spots of intradermal injections.

Photo showing intradermal injection spots in the back of a mouse after euthanasia and dissection and Quantification of Evans Blue extravasation at the cutaneous spots. p=0.31.

**Supplemental Figure 3: The increase of the HUVEC permeability for low molecular Weight molecules *in vitro* is not linked to the caveolin 1 pathway**

**A**: Western Blot carried out on HUVEC cell lysates after 1 hour of stimulation by the sera of MCD or HV patients

**B:** Semi-quantitative measurement of the expression of different proteins by Gene Tools software (Syngene). p=1

**C**: Transfection of HUVEC by caveolin 1 siRNA or control siRNA then measurement of the Sodium Fluorescein (NaF) passage through a semipermeable membrane of the Transwell system (pore: 0.4 μm diameter) after stimulation of HUVECS by sera from MCD or HV patients at 15 (A), 30 (B), 45 (C) minutes and 1 hour (D) after the start of stimulation. The results are expressed as the ratio of the fluorescence intensity between the lower and upper chambers. *: p=0.029

**Supplemental Figure 4: The cleavage of ENaC in vitro is not increased by MCD sera.**

**A**: Western Blot carried out with anti-ENaC-α on HUVEC cell lysates after 1 hour of stimulation by the sera of MCD or HV patients

**B**: Western Blot carried out with anti-ENaC-β on HUVEC cell lysates after 1 hour of stimulation by the sera of MCD or HV patients

**C**: Western Blot carried out with anti-ENaC-γ on HUVEC cell lysates after 1 hour of stimulation by the sera of MCD or HV patients

**
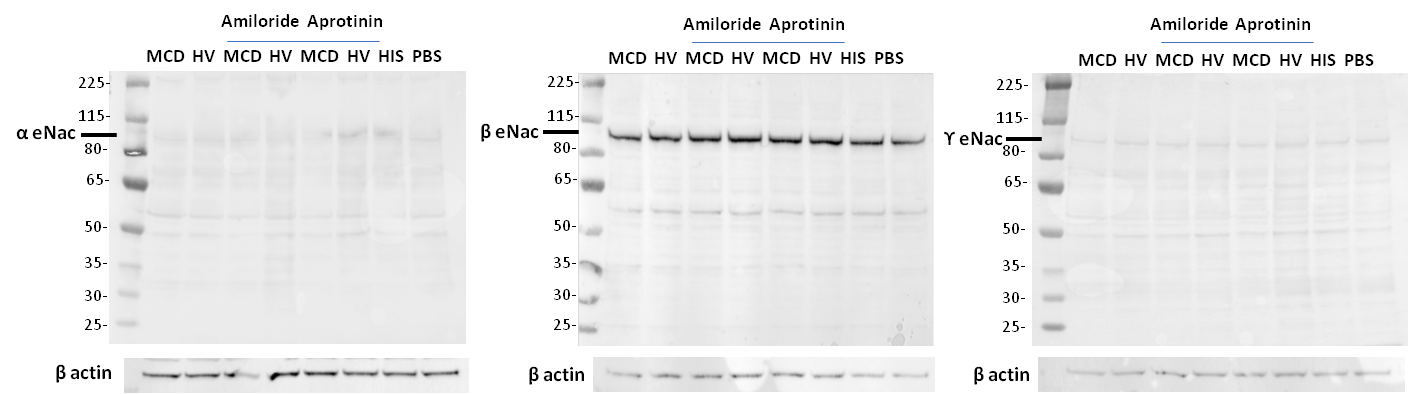
**

**Supplemental references**

[S1] Schork A, Woern M, Kalbacher H, Voelter W, Nacken R, Bertog M, et al. Association of Plasminuria with Overhydration in Patients with CKD. Clin J Am Soc Nephrol. 2016 May 6;11(5):761–9.

[S2] Kusche-Vihrog K, Tarjus A, Fels J, Jaisser F. The epithelial Na+ channel: a new player in the vasculature. Curr Opin Nephrol Hypertens. 2014 Mar;23(2):143–8.

[S3] Jeggle Pia, Callies Chiara, Tarjus Antoine, Fassot Celine, Fels Johannes, Oberleithner Hans, et al. Epithelial Sodium Channel Stiffens the Vascular Endothelium In Vitro and in Liddle Mice. Hypertension. 2013 May 1;61(5):1053–9.

[S4] Kleyman T, Cragoe E. Amiloride and its analogs as tools in the study of ion transport. J Membrane Biol 1988;105–121.

[S5] Jaffe EA, Nachman RL, Becker CG, Minick CR. Culture of Human Endothelial Cells Derived from Umbilical Veins. J Clin Invest. 1973 Nov;52(11):2745–56.

[S6] Kustermann S, Manigold T, Ploix C, Skubatz M, Heckel T, Hinton H, et al. A Real-time Impedance-Based Screening Assay for Drug-Induced Vascular Leakage. Toxicol Sci. 2014 Apr 1;138(2):333–43.

[S7] Hahn CS, Scott DW, Xu X, Roda MA, Payne GA, Wells JM, et al. The matrikine N-α-PGP couples extracellular matrix fragmentation to endothelial permeability. Sci Adv [Internet]. 2015 Apr 24 [cited 2016 Feb 8];1(3). Available from: http://www.ncbi.nlm.nih.gov/pmc/articles/PMC4517288/

[S8] Bossi F, Fischetti F, Pellis V, Bulla R, Ferrero E, Mollnes TE, et al. Platelet-Activating Factor and Kinin-Dependent Vascular Leakage as a Novel Functional Activity of the Soluble Terminal Complement Complex. J Immunol. 2004 Dec 1;173(11):6921–7.

[S9] Eriksson A, Cao R, Roy J, Tritsaris K, Wahlestedt C, Dissing S, et al. Small GTP-Binding Protein Rac Is an Essential Mediator of Vascular Endothelial Growth Factor-Induced Endothelial Fenestrations and Vascular Permeability. Circulation. 2003 Mar 25;107(11):1532–8.
